# Identification of Anti-Inflammatory Compounds from *Peucedanum praeruptorum* Roots by Using Nitric Oxide-Producing Rat Hepatocytes Stimulated by Interleukin 1β

**DOI:** 10.3390/molecules28135076

**Published:** 2023-06-28

**Authors:** Hiromu Ozaki, Yuto Nishidono, Airi Fujii, Tetsuya Okuyama, Kaito Nakamura, Takanori Maesako, Saki Shirako, Richi Nakatake, Ken Tanaka, Yukinobu Ikeya, Mikio Nishizawa

**Affiliations:** 1 Department of Biomedical Sciences, College of Life Sciences, Ritsumeikan University, Kusatsu 525-8577, Shiga, Japansshirako@fc.ritsumei.ac.jp (S.S.); 2 College of Pharmaceutical Sciences, Ritsumeikan University, Kusatsu 525-8577, Shiga, Japan; nisidono@fc.ritsumei.ac.jp (Y.N.); ktanaka@fc.ritsumei.ac.jp (K.T.); 3 Research Organization of Science and Technology, Ritsumeikan University, Kusatsu 525-8577, Shiga, Japan; 4 Department of Surgery, Kansai Medical University, Hirakata 573-1010, Osaka, Japan; okuyamat@hirakata.kmu.ac.jp (T.O.); nakatakr@hirakata.kmu.ac.jp (R.N.); 5 Faculty of Pharmacy, Daiichi University of Pharmacy, Fukuoka 815-8511, Fukuoka, Japan; y-ikeya@daiichi-cps.ac.jp; 6 Asia-Japan Research Institute, Ritsumeikan Asia-Japan Research Organization, Ritsumeikan University, Ibaraki 567-8570, Osaka, Japan

**Keywords:** pyranocoumarin, Kampo medicine, inflammation, nitric oxide, hepatocyte, bioassay-guided isolation

## Abstract

The roots of *Peucedanum praeruptorum* Dunn and *Angelica decursiva* Franchet et Savatier are designated *Zenko*, which is a crude drug defined by the Japanese Pharmacopoeia. This crude drug is used as an antitussive and an expectorant and is included in the Kampo formula *Jinsoin*, which improves cough, fever, and headache. Although the anti-inflammatory effects of this crude drug have been determined, the constituents responsible for this effect remain unknown. To investigate biologically active compounds, rat hepatocytes were used, which produce proinflammatory mediator nitric oxide (NO) in response to proinflammatory cytokine interleukin 1β (IL-1β). A methanol extract of *P. praeruptorum* roots, which suppressed IL-1β-induced NO production, was fractionated into three crude fractions (ethyl acetate (EtOAc)-soluble, *n*-butanol-soluble, and water-soluble fractions) based on hydrophobicity. The EtOAc-soluble fraction markedly inhibited NO production. After this fraction was purified, three biologically active compounds were identified as praeruptorins A, B, and E, the contents of which were high. A comparison of their activities indicated that praeruptorin B exhibited the highest potency to inhibit NO production by decreasing inducible NO synthase expression and suppressed the expression of mRNAs encoding proinflammatory cytokines. Collectively, the three praeruptorins may primarily contribute to the anti-inflammatory effects of *P. praeruptorum* roots.

## 1. Introduction

When investigating the effects of crude drugs used in traditional Asian medicine, such as Japanese Kampo medicine, it is essential to identify their principal constituents, which are biologically active and show pharmacological activities. Although most principal constituents are relatively abundant, they are often not well studied. Principal constituents from many compounds in crude drugs can be identified through bioassay-guided procedures involving the purification and identification of active constituents.

To identify anti-inflammatory constituents, we used primary cultured rat hepatocytes, which produce proinflammatory mediator nitric oxide (NO) in response to proinflammatory cytokine interleukin 1β (IL-1β) [1,2]. NO is synthesized by inducible nitric oxide synthase (iNOS, also known as NOS2) in hepatocytes and macrophages [3]. *iNOS* mRNA expression increased 2 h after IL-1β was added, peaked at 6 h, and then decreased, whereas iNOS protein increased after IL-1β addition, and NO production correlated with the iNOS protein level [4].

Anti-inflammatory compounds and drugs inhibit IL-1β-induced NO production in hepatocytes and in lipopolysaccharide (LPS)-treated macrophages, e.g., RAW264.7 cells. When NO production in the presence of a drug is compared with that in the presence of IL-1β alone, the potency of NO suppression can be calculated and expressed as a half-maximal inhibitory concentration (IC_50_) value [3,5]. Principal constituents in a crude drug have been identified by comparing the IC_50_ values of the compounds [6,7,8]. The proinflammatory genes include iNOS, proinflammatory cytokine, and chemokine genes, most of which are regulated by transcription factor NF-κB [9]. We report that almost all the compounds that suppressed NO production inhibited the mRNA expression of proinflammatory genes [10,11,12].

*Peucedanum praeruptorum* Dunn (Umbelliferae) and *Angelica decursiva* Franchet et Savatier (Umbelliferae) grow in China, and their roots are designated *Zenko*, which is a crude drug defined by the Japanese Pharmacopoeia [13]. Due to its anti-inflammatory effect, this crude drug is used as an antitussive and an expectorant and is included in *Jinsoin*, which is a Kampo formula used to improve symptoms such as cough, fever, and headache. Although *A. decursiva* and *P. praeruptorum* roots possess many coumarins, the former exclusively contains nodakenin [14], and the latter exclusively contains praeruptorin [15,16].

In the extracts of *P. praeruptorum* roots, many pyranocoumarins (e.g., praeruptorin), furanocoumarins, and a polyacetylene (i.e., falcarindiol) were identified [15,16]. Among the 16 compounds identified in *P. praeruptorum* roots by Lee et al., only praeruptorin A and falcarindiol decreased NO production in the mouse macrophage line RAW 264.7 in the presence of LPS [17]. Two other reports stated that praeruptorins, including praeruptorin A, reduced NO production in RAW264.7 cells [18,19]. Because the IC_50_ values were not provided in either report, the principal constituents responsible for the suppression of NO production remained unassigned.

In this study, the gene expression profiles of primary cultured rat hepatocytes were characterized in the presence of IL-1β. Then, we analyzed constituents from *P. praeruptorum* roots and searched for anti-inflammatory constituents by monitoring the suppression of IL-1β-induced NO production using this ex vivo hepatocyte system (NO assay). By bioassay-guided purification, we tried to identify the principal constituents in *P. praeruptorum* roots. Finally, the activity-based screening of constituents in crude drugs is discussed.

## 2. Results

### 2.1. Gene Expression in the Presence of IL-1β in Rat Hepatocytes

**Figure 1:**
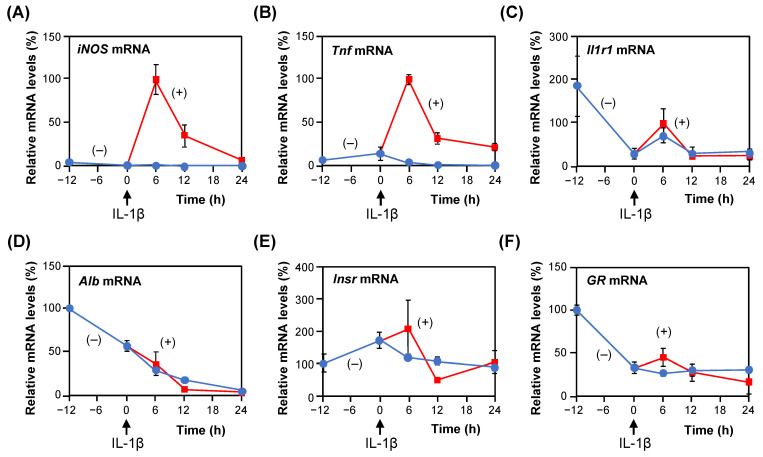
The mRNA expression in primary cultured rat hepatocytes after the addition of IL-1β. On day 0, rat hepatocytes were seeded, and total RNA was extracted after the second medium change (–12 h before IL-1β addition). If needed, rat IL-1β was added to the medium of the hepatocytes to a final concentration of 1 nM (0 h). The hepatocytes were further incubated in the absence (–) or presence (+) of IL-1β. Total RNA was extracted at 0, 6, 12, and 24 h after IL-1β addition and subjected to RT–qPCR to estimate the levels of mRNAs encoding rat iNOS, TNF-α (Tnf), IL1R1 (Il1r1), albumin (Alb), insulin receptor (Insr), and glucocorticoid receptor (GR). The levels of mRNA were measured using eukaryotic elongation factor 1α (*EF-1α*, *Eef1a1*) mRNA as an internal control and were normalized to *EF-1α* mRNA. Relative mRNA levels are presented as the mean ± standard deviation (SD; *n* = 3) of the resulting percentage: (**A**) *iNOS* mRNA; (**B**) *Tnf* mRNA; (**C**) *Il1r1* mRNA; (**D**) *Alb* mRNA; (**E**) *Insr* mRNA; and (**F**) *GR* mRNA. The mRNA level at –12 h before IL-1β addition (**D**–**F**) or at 6 h after IL-1β addition (**A**–**C**) was set as 100%.

To investigate the profiles of mRNA expression in primary cultured hepatocytes, total RNA was extracted from the hepatocytes during incubation and analyzed by quantitative reverse transcription–polymerase chain reaction (RT–qPCR). When IL-1β was added to the medium (0 h on day 1), *iNOS* mRNA increased, peaked at 6 h, and then decreased at 12 h after IL-1β addition (Figure 1), as previously observed [4]. Similar induction was observed in tumor necrosis factor-α (TNF-α). The *iNOS* and *Tnf* genes are involved in inflammation and are under the control of the IL-1 signaling pathway mediated by IL-1 receptor type 1 (IL1R1) [20,21,22]. Although *Il1r1* mRNA decreased after cell seeding, it was induced and peaked 6 h after IL-1β addition.

As previously reported, NO production increased linearly with time, and a logarithmic increase was observed approximately 8 h after IL-1β addition [4]. Anti-inflammatory compounds and drugs suppress proinflammatory genes to reduce the production of proinflammatory mediators, such as NO, cytokines, and chemokines. Hereafter, NO levels in the medium were measured 8 h after IL-1β addition on day 1 to calculate IC_50_ values of suppressing NO production by a compound.

The expression of the albumin gene, which encodes the precursor of the most abundant protein in the blood, decreased after cell seeding (Figure 1), as previously observed [23]. The mRNA levels of serine dehydratase, which is an abundant enzyme in the liver of rodents and plays an important role in gluconeogenesis [24], decreased to less than 3% at 6 h after IL-1β addition when the mRNA level at −12 h was set as 100%. In contrast, the mRNA levels of the insulin receptor (Insr), which plays a central role in insulin-mediated regulation of cellular metabolism and growth of hepatocytes [25], did not markedly change. The mRNA levels of the glucocorticoid receptor (GR), which is also known as nuclear receptor subfamily 3, group C, member 1 (Nr3c1), did not significantly change. When synthetic glucocorticoid dexamethasone was added to the medium, it significantly suppressed NO production with an IC_50_ value of 18.6 nM. Taken together, the results indicate that the hepatocytes maintained their functions, such as glucose metabolism and inflammatory responses.

### 2.2. Crude Fractionation of P. praeruptorum Root Extract

*P. praeruptorum* roots were extracted with methanol, as previously described [10]. The resultant extract was sequentially separated based on hydrophobicity into the following crude fractions: EtOAc-soluble (fraction A); *n*-butanol-soluble (fraction B); and water-soluble fractions (fraction C).

**Figure 2:**
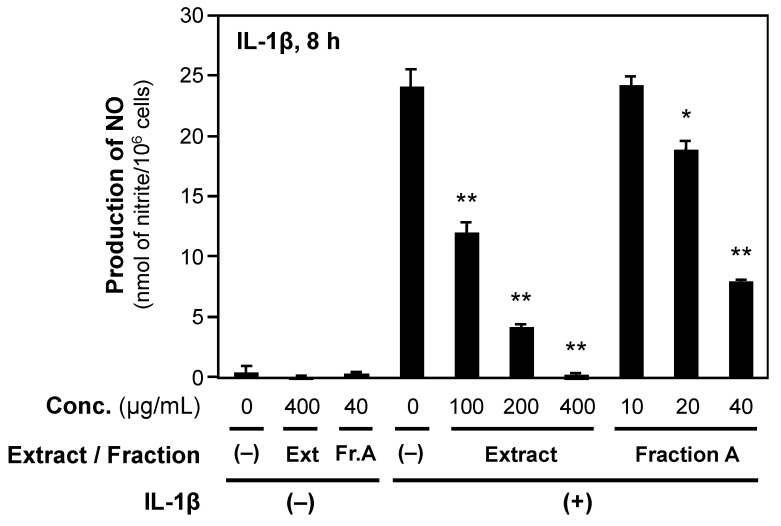
Effects of *P. praeruptorum* root extract and fraction A on NO production in hepatocytes. Hepatocytes were treated with 1 nM IL-1β in the presence or absence of *P. praeruptorum* root extract or fraction A (Fr.A) for 8 h. The levels of nitrite (a major metabolite of NO) in the medium were measured and depicted as the mean ± SD (*n* = 3 dishes). * *p* < 0.05 and ** *p* < 0.01 versus IL-1β alone.

Using rat hepatocytes, we evaluated the ability to inhibit NO production in the presence of IL-1β. Methanol extract and fraction A significantly inhibited IL-1β-induced NO production (Figure 2). In contrast, fraction C weakly suppressed NO production. Lactose dehydrogenase (LDH) activities in the medium of hepatocytes treated with methanol extract and each crude fraction were low (less than 5% of the activity of a whole cell extract), suggesting that neither methanol extract nor crude fraction was cytotoxic to hepatocytes.

Next, the IC_50_ values of NO production were calculated (Table 1). Fraction A possessed the highest potency among the crude fractions. It was expected that fraction A may include the constituents that suppress NO production in IL-1β-treated hepatocytes.

### 2.3. Identification of Biologically Active Compounds in Fraction A

**Scheme 1:**
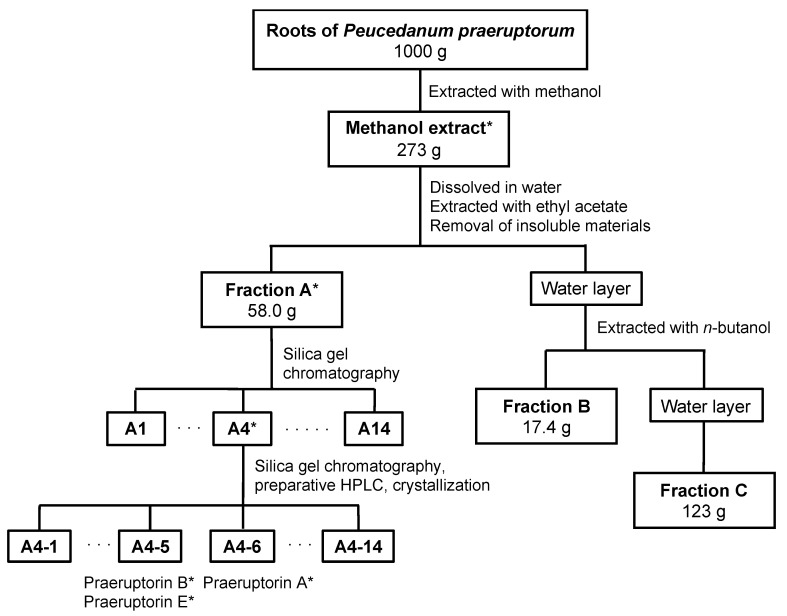
A flowchart of NO-assay-guided purification of the constituents of *P. praeruptorum* roots. The methanol extract was fractionated into fractions A (hydrophobic fraction), B (amphipathic fraction), and C (hydrophilic fraction). Compounds included in the relevant subfraction are shown. * NO production was inhibited in hepatocytes.

**Figure 3:**
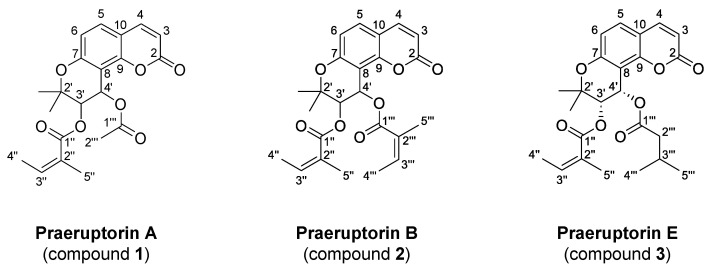
Chemical structures of praeruptorins A, B, and E.

To purify and identify the constituents of the *P. praeruptorum* root extract, fraction A was fractionated by silica gel column chromatography into subfractions A1 to A14 (Scheme 1). When these subfractions were evaluated by NO assays, subfraction A4 exhibited the highest activity against NO production in hepatocytes. Therefore, we tried to purify biologically active compounds from this subfraction. Three compounds, **1** to **3,** were isolated according to Scheme 1 and identified by nuclear magnetic resonance (NMR) spectroscopy and mass spectrometry (MS) analyses (Figure 3).

Compound **1** showed the following characteristics: White crystals; [*α*${]}_{D}^{20}$ +22.1 (*c* 0.147, CHCl_3_); melting point (mp), 136–137 °C; ^1^H NMR (400 MHz, CDCl_3_, ppm) *δ* 7.59 (1H, d, *J* = 9.6 Hz, H-4), 7.34 (1H, d, *J* = 8.8 Hz, H-5), 6.79 (1H, d, *J* = 8.8 Hz, H-6), 6.58 (1H, d, *J* = 5.2 Hz, H-3’), 6.23 (1H, d, *J* = 9.6 Hz, H-3), 6.12 (1H, br q, *J* = 7.2 Hz, H-3’’), 5.39 (1H, d, *J* = 5.2 Hz, H-4’), 2.10 (3H, s, H-2’’’), 1.95 (3H, br d, *J* = 7.2 Hz, H-4’’), 1.85 (3H, br s, H-5’’), 1.46 (3H, s, 2’-CH_3_), 1.42 (3H, s, 2’-CH_3_); and ^13^C NMR (100 MHz, CDCl_3_, ppm) δ 169.9 (C-1’’’), 166.5 (C-1’’), 160.0 (C-2), 156.8 (C-7), 154.1 (C-9), 143.4 (C-4), 140.0 (C-3’’), 129.2 (C-5), 127.0 (C-2’’), 114.4 (C-6), 113.3 (C-3), 112.6 (C-10), 107.1 (C-8), 77.8 (C-2’), 69.8 (C-4’), 61.1 (C-3’), 25.0 (2’-CH_3_), 23.1 (2’-CH_3_), 20.8 (C-2’’’), 20.6 (C-5’’), 15.9 (C-4’’). This compound was identified as praeruptorin A on the basis of ^1^H NMR and ^13^C NMR spectral analysis by comparison with previously published results [15]. Its melting point agreed with that of published data of (+)-praeruptorin A [26].

Compound **2** showed the following characteristics: White powder; [*α*${]}_{D}^{20}$ +12.4 (*c* 0.128, CHCl_3_); electron spray ionization (EI)–MS *m*/*z* (%): 426 (M^+^, 9.3), 327 [M^+^-CH_3_CH=CH(CH_3_)-COO, 50], 227 (12), 83 [CH_3_CH=CH(CH_3_)-C≡O^+^, 100], 55 (18).; high-resolution (HR) EI–MS, *m*/*z* 426.1683 [M]^+^ (calculated for C_24_H_26_O_7_; 426.1679); ^1^H NMR (400 MHz, CDCl_3_, ppm) *δ* 7.58 (1H, d, *J* = 9.6 Hz, H-4), 7.34 (1H, d, *J* = 8.8 Hz, H-5), 6.80 (1H, d, *J* = 8.8 Hz, H-6), 6.69 (1H, d, *J* = 5.2 Hz, H-3’), 6.21 (1H, d, *J* = 9.6 Hz, H-3), 6.12 (1H, br q, *J* = 7.2 Hz, H-3’’’), 6.02 (1H, br q, *J* = 7.2 Hz, H-3’’), 5.44 (1H, d, *J* = 5.2 Hz, H-4’), 1.97 (3H, br d, *J* = 7.2 Hz, H-4’’), 1.95 (3H, br d, *J* = 7.2 Hz, H-4’’’), 1.84 (3H, br s, H-5’’), 1.81 (3H, br s, H-5’’’), 1.48 (3H, s, 2’-CH_3_), 1.44 (3H, s, 2’-CH_3_); and ^13^C NMR (100 MHz, CDCl_3_, ppm) *δ* 166.6 (C-1’’’), 166.3 (C-1’’), 159.9 (C-2), 156.8 (C-7), 154.1 (C-9), 143.4 (C-4), 140.0 (C-3’’’), 138.6 (C-3’’), 129.3 (C-5), 127.4 (C-2’’’), 127.0 (C-2’’), 114.4 (C-6), 113.3 (C-3), 112.5 (C-10), 107.6 (C-8), 77.5 (C-2’), 70.2 (C-4’), 60.2 (C-3’), 25.5 (2’-CH_3_), 22.6 (2′-CH_3_), 20.5 (C-5’’’), 20.4 (C-5’’), 15.9 (C-4’’’), 15.7 (C-4’’). This compound was identified as praeruptorin B on the basis of ^1^H NMR and ^13^C NMR spectral analysis by comparison with previously published results [15].

Compound **3** showed the following characteristics: White powder; [*α*${]}_{D}^{20}$ +28.7 (*c* 0.124, CHCl_3_); ^1^H NMR (400 MHz, CD_3_OD, ppm) *δ* 7.58 (1H, d, *J* = 9.6 Hz, H-4), 7.34 (1H, d, *J* = 8.8 Hz, H-5), 6.79 (1H, d, *J* = 8.8 Hz, H-6), 6.61 (1H, d, J = 4.8 Hz, H-3’), 6.22 (1H, d, *J* = 9.6 Hz, H-3), 6.11 (1H, br q, *J* = 8.8 Hz, H-3’’), 5.39 (1H, d, *J* = 4.8 Hz, H-4’), 2.10–2.30 (2H, m, H-2’’’), 1.95 (3H, br d, *J* = 8.8 Hz, H-4’’), 1.86 (3H, br s, H-5’’), 1.46 (3H, s, 2’-CH_3_), 1.43 (3H, s, 2’-CH_3_), 1.16–1.24 (1H, m, H-3’’’), 0.94 (3H, br d, *J* = 6.4 Hz, H-4’’’), 0.89 (3H, br d, *J* = 6.4 Hz, H-5’’’); and ^13^C NMR (100 MHz, CD_3_OD, ppm) *δ* 171.9 (C-1’’’), 166.5 (C-1’’), 159.9 (C-2), 156.7 (C-7), 154.1 (C-9), 143.3 (C-4), 139.9 (C-3’’), 129.9 (C-5), 127.0 (C-2’’), 114.4 (C-6), 113.3 (C-3), 112.5 (C-10), 107.4 (C-8), 77.7 (C-2’), 70.1 (C-4′), 60.6 (C-3′), 43.3 (C-2’’’), 25.5 (C-3’’’), 25.3 (2’-CH_3_), 22.8 (2’-CH_3_), 22.6 (C-4’’’), 22.5(C-5’’’), 20.6 (C-5’’), 15.9 (C-4’’). This compound was identified as (+)-praeruptorin E on the basis of ^1^H NMR and ^13^C NMR spectral analysis by comparison with previously published results [15,16].

### 2.4. NO Production Is Suppressed by Compounds in P. praeruptorum Fraction A

**Figure 4:**
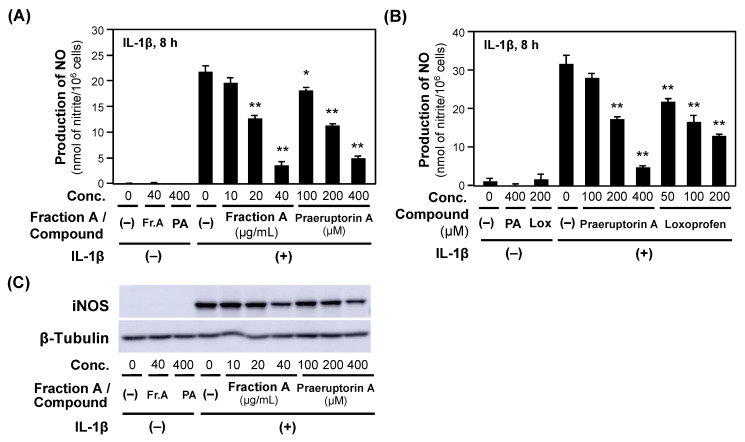
Effects of fraction A and praeruptorin A on NO production and iNOS expression in hepatocytes. (**A**) Effects of fraction A and praeruptorin A on NO production. Hepatocytes were treated with 1 nM IL-1β ± fraction A (Fr.A) or praeruptorin A (PA) for 8 h. The levels of nitrite in the medium were measured and are shown as the mean ± SD (*n* = 3). * *p* < 0.05 and ** *p* < 0.01 versus IL-1β alone. (**B**) Comparison of the effects of praeruptorin A and loxoprofen on NO production. Loxoprofen (Lox) was added to the medium as a positive control. Similar to (**A**), the NO concentration was measured and presented. ** *p* < 0.01 versus IL-1β alone. (**C**) Effects of fraction A and praeruptorin A on iNOS expression. Cell extracts were prepared from hepatocytes treated in (**A**). The extracts were analyzed by Western blotting to detect and visualize iNOS (130 kDa) and β-tubulin (55 kDa; internal control).

Next, we examined whether these three pyranocoumarins derived from fraction A suppress NO production in IL-1β-treated hepatocytes. First, praeruptorin A was examined because it is a major constituent in *P. praeruptorum* root extract. Similar to fraction A, when praeruptorin A was added to the medium, it decreased NO production in a concentration-dependent manner (Figure 4) without showing cytotoxicity to hepatocytes (i.e., less than 5% of LDH activity of a whole cell extract). Furthermore, Western blot analysis was performed with the cell extracts prepared from the hepatocytes that were treated with praeruptorin A, and the results indicated that praeruptorin A reduced levels of iNOS protein in hepatocytes. Compared with the effect of the positive control loxoprofen, which is a nonsteroidal anti-inflammatory drug [3], praeruptorin A suppressed NO production less efficiently (Figure 4).

Next, praeruptorins B and E were examined by NO assays. The potencies of the three praeruptorins are summarized in Table 2, and the results suggest that praeruptorin B shows the greatest ability to suppress NO production. These data imply that the compounds purified from faction A possess the ability to inhibit NO production in IL-1β-treated hepatocytes.

### 2.5. Analysis of the Compounds in Fraction A of P. praeruptorum Roots

Then, we measured the content of each praeruptorin by high-performance liquid chromatography (HPLC) analysis, as described in the Materials and Methods section. As shown in Table 2, the constituent of *P. praeruptorum* roots with the greatest concentration was praeruptorin A. In fraction A, the three praeruptorins comprised 40.8% of the fraction.

**Figure 5:**
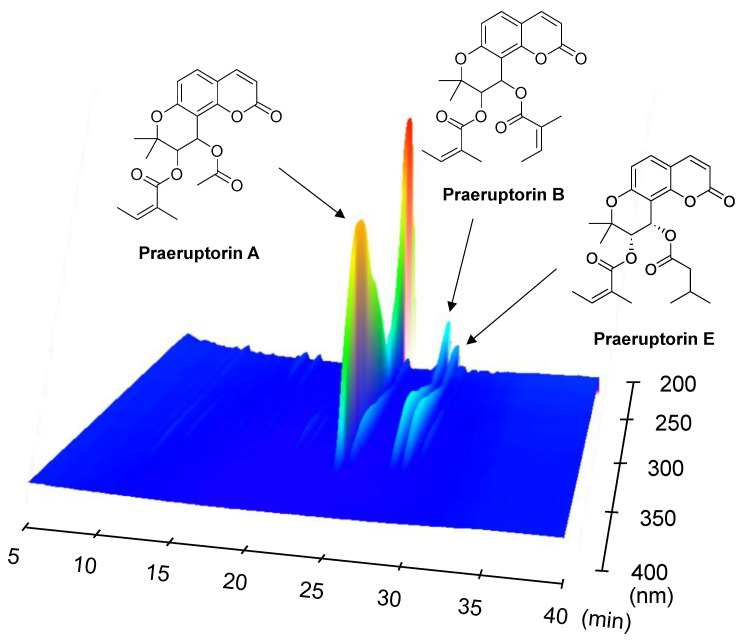
3D-HPLC profiles of fraction A of *P. praeruptorum* root extract. Fraction A was analyzed as described in the Materials and Methods section. *x*-axis, retention time (min); *y*-axis, wavelength (nm); *z*-axis, absorbance. Compounds corresponding to the peaks are indicated.

To confirm the results regarding the content, 3D-HPLC fingerprints of fraction A were analyzed. The data indicated that praeruptorins A, B, and E were the top three constituents that absorbed UV light in fraction A of the *P. praeruptorum* root extract (Figure 5).

Collectively, the three pyranocoumarins that were purified from *P. praeruptorum* roots by monitoring NO assays possessed the ability to suppress NO production in rat hepatocytes. It is highly expected that these pyranocoumarins exhibit anti-inflammatory effects, similar to previously published reports [5,10].

### 2.6. Praeruptorins A and B Decrease the mRNA Levels of Proinflammatory Genes

**Figure 6:**
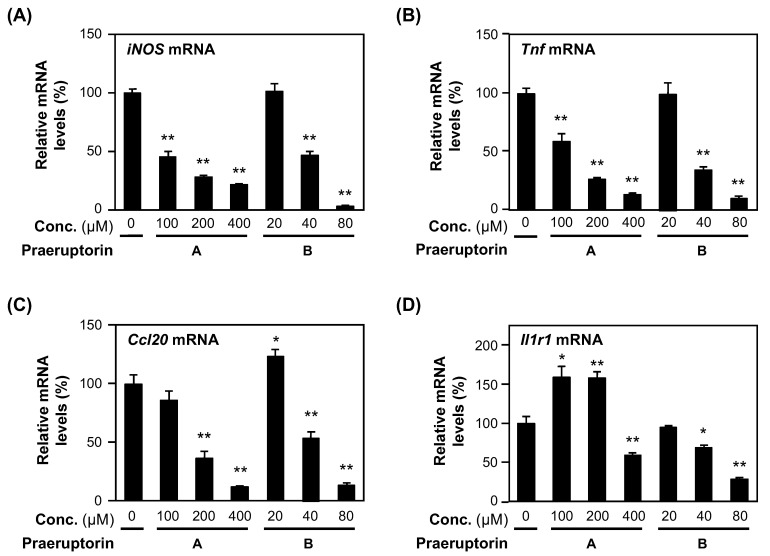
Effects of praeruptorin A and B on the mRNA expression of proinflammatory genes. Each compound and IL-1β were added to the medium of rat hepatocytes and further incubated for 4 h. Then, total RNA was extracted and subjected to RT–qPCR. The levels of each mRNA were measured in triplicate and normalized to the *EF-1α* mRNA level: (**A**) *iNOS* mRNA; (**B**) *Tnf* mRNA; (**C**) *Ccl20* mRNA; and (**D**) *Il1r1* mRNA. Relative mRNA levels are shown as the mean ± SD (*n* = 3) of the resulting percentage. In the presence of IL-1β alone, the mRNA level measured was set as 100%. When IL-1β was not added or when each compound alone was added to the medium, amplification was not detected. * *p* < 0.05 and ** *p* < 0.01 versus IL-1β alone.

We examined whether these biologically active pyranocoumarins from fraction A affect the mRNA expression of proinflammatory genes. Praeruptorin A (the highest content) and B (the highest activity) were selected, and the mRNA levels were measured by RT–qPCR using total RNA from hepatocytes, each treated with praeruptorin and IL-1β. The obtained mRNA levels were normalized to those of mRNA encoding eukaryotic elongation factor (EF-1α, an internal control) to provide relative mRNA levels. As expected from the data about NO production and the expression of iNOS protein, praeruptorin A decreased the *iNOS* mRNA levels (Figure 6). In contrast, praeruptorin B decreased *iNOS* mRNA levels at a much lower concentration than that of praeruptorin A.

Finally, the relative levels of mRNA encoding inflammatory cytokines (i.e., TNF-α and IL-6), chemokine (C-C motif) ligand 20 (CCL20), and IL1R1 were investigated (Figure 6). Praeruptorin A reduced the mRNA levels of *Tnf*, *Ccl20*, and *Il1r1*, and praeruptorin B also suppressed the expression of these mRNAs. Compared to praeruptorin A, praeruptorin B decreased the levels at a much lower concentration. These data suggest that praeruptorins A and B suppress the expression of the genes involved in inflammation.

## 3. Discussion

In this study, the principal constituents responsible for the anti-inflammatory effects of *P. praeruptorum* roots were identified by NO assays using IL-1β-treated rat hepatocytes. The mRNA expression profiles of the hepatocytes could induce proinflammatory genes, which synthesize proinflammatory mediators, i.e., NO, cytokines, and chemokines (Figure 1). NO was logarithmically increased at 8 h after IL-1β addition on day 1 [4]. IL-1β-induced production of NO, cytokines, and chemokines is inhibited by an anti-inflammatory compounds ex vivo system that is suitable for evaluating anti-inflammatory effects and isolating biologically active compounds, i.e., praeruptorins, in crude drugs because it is easy to perform measurements with by taking the medium (e.g., NO and cytokines) or the cells (e.g., total RNA and cell extracts).

To identify the active fraction that inhibited NO production in hepatocytes, three pyranocoumarins were specified as principal constituents in a methanol extract of *P. praeruptorum* roots (Scheme 1). Among many compounds previously reported [15,16], praeruptorins A, B, and E were identified as principal constituents of *P. praeruptorum* roots by this bioassay-guided isolation method. Praeruptorin A decreased NO production in LPS-treated RAW 264.7 cells [17,18]; however, compared to praeruptorin A, praeruptorin B exhibited 4.8-fold higher potency to suppress NO production in hepatocytes based on IC_50_ values (Table 2). In addition, falcarindiol suppressed NO production in RAW 264.7 cells [17]. However, its content in *P. praeruptorum* roots was very low based on 3D-HPLC analysis (Figure 5), implying that falcarindiol may contribute little to the anti-inflammatory effects of *P. praeruptorum* roots.

The structure–activity relationship of praeruptorins in the suppression of NO production should be considered. This study indicated that the optical rotation of compound **2** was [*α*${]}_{D}^{20}$ +12.4 (*c* 0.128, CHCl_3_), whereas Song et al. reported that the optical rotations of (+)- and (–)-praeruptorin B were [*α*${]}_{D}^{20}$ +37 and –36 (*c* 1.0, CDCl_3_), respectively [16]. It cannot be ruled out that compound **2** is a mixture of (+)- and (–)-praeruptorin B.

Praeruptorins A, B, and E are khellactones with an angelyol group at C-3’ and a different substituent at C-4’: acetyl (praeruptorin A), angeloyl (praeruptorin B), and isovaleryl groups (praeruptorin E) (Figure 3). The IC_50_ values obtained in this study were as follows: praeruptorin A > praeruptorin B > praeruptorin E (Table 2). The suppression activity to reduce NO production was high when an angeloyl group was a substituent at C-4’, such as praeruptorin B, whereas it was lower when an acetyl group was a substituent at C-4’, such as praeruptorin A. It is implied that the substituent at C-4’ may be involved in activity to reduce NO production in hepatocytes. Lee et al. reported the structure–activity relationship using several khellactones with different substituents at C-3’ and C-4’ from the root and rhizome of *Glehnia littoralis* in LPS-treated RAW264.7 cells [27]. The IC_50_ value of 3’-senecioyl-4’-acetylkhellactone was higher than that of 3’,4’-disenecioylkhellactone, and the IC_50_ value of 3’-isovaleryl-4’-acetylkhellactone was higher than that of both 3’,4’-diisovalerylkhellactone and 3’-isovaleryl-4’-senecioylkhellactone. These results may support our hypothesis that the substituent at C-4’ may be involved in the activity. To elucidate the structure–activity relationship of praeruptorins, more enantiomers of praeruptorins and pyranocoumarin derivatives should be investigated by NO assays in the future.

Accumulating data on the IC_50_ values of many constituents isolated from crude drugs have shown that many compounds suppress NO production in rat hepatocytes. Among the three pyranocoumarins, praeruptorin B exhibited the highest potency in the suppression of NO production, with an IC_50_ value of 43.5 μM (Table 2). The compounds that showed comparative IC_50_ values for praeruptorin B are nectandrin B (43.4 μM), a lignan from the heartwood of *Guaiacum officinale* or *G. sanctum* [28]; umbelliferone (42.0 μM), a hydroxycoumarin from the root and rhizome of *Glehnia littoralis* [29]; and liquiritigenin (41.2 μM), a flavonoid from the root and stolon of *Glycyrrhiza uralensis* [30]. In contrast, praeruptorin A, identified as the major constituent by HPLC and 3D-HPLC analyses (Table 2, Figure 5), showed a much higher IC_50_ value (208 μM), which is close to that of gallic acid (212 μM) [31]. Furthermore, it is mentioned that anti-inflammatory effects are involved in anti-aging and antidiabetic activities [32,33].

Rat hepatocytes are feasible for studies because they provide an ex vivo system to maintain the NO-producing feature of hepatocytes in vivo. Previously, we compared the profiles of NO production and iNOS expression in rat hepatocytes with those in macrophage lines, including RAW264.7 cells [3]. The anti-inflammatory effects of four antipyretic analgesic drugs were interchangeably evaluated by rat hepatocytes and RAW264.7 cells, although there were slight differences in their IC_50_ values [3]. LPS-induced NO production in RAW264.7 cells may also be applied for bioassay-guided isolation of anti-inflammatory constituents from crude drugs. These approaches clarify the principal anti-inflammatory constituents that are not well studied. Inflammation is involved in pathophysiological conditions in many diseases, including infection, metabolic disease, and cancer. For example, *P. praeruptorum* root extract was reported to exhibit an anticancer effect on the growth of cancer cell lines [34]. The bioassay-guided isolation of active compounds facilitates studies on pharmacognosy and pharmacology, including analyses of their chemical structures and pharmacological activities.

## 4. Materials and Methods

### 4.1. General Experimental Procedures

Silica gel column chromatography was performed with Silica gel 60 (20–230 mesh; Nacalai Tesque Inc., Kyoto, Japan) or Wakogel C-300 HG (Fujifilm Wako Pure Chemical Corporation, Osaka, Japan). Precoated TLC was performed on Silica gel 60 F_254_ plates (Fujifilm Wako Pure Chemical Corporation). NMR spectra were recorded using a JNM-ECS400 NMR spectrometer (JEOL Ltd., Akishima, Tokyo, Japan), which was operated at 400 MHz (^1^H) and 100 MHz (^13^C). Tetramethylsilane (internal standard of NMR spectrometry), deuterated chloroform (CDCl_3_), and deuterated methanol (CD_3_OD) were obtained from Euriso-Top (Saint-Aubin, France). Optical rotations of compounds were measured on a DIP-1000 polarimeter (JASCO Corporation, Hachioji, Tokyo, Japan). EI–MS spectra were obtained with a JMS-700 MStation mass spectrometer (JEOL Ltd.). ChemDraw software (PerkinElmer Inc., Waltham, MA, USA) was used to draw chemical structures.

### 4.2. Plant Material

*P. praeruptorum* Thunberg roots that were collected from Zhejiang Province, China, were obtained from Tochimoto Tenkaido Co., Ltd., (Osaka, Japan). Dr. Yutaka Yamamoto (Tochimoto Tenkaido Co., Ltd.) authenticated them as *Zenko*. The voucher specimen was deposited in the Ritsumeikan Herbarium of Pharmacognosy, Ritsumeikan University, under the code number RIN-PP-34.

### 4.3. Crude Fractionation of a Methanol Extract

Dried *P. praeruptorum* roots (1.00 kg) were pulverized and extracted with methanol (5 L) under reflux three times and evaporated under vacuum, yielding 273 g (27.3%). Methanol extract of *P. praeruptorum* roots was dissolved in water and extracted by sequential partitioning with EtOAc and *n*-butanol. Each extract was then concentrated under reduced pressure to generate the EtOAc-soluble fraction (fraction A), *n*-butanol-soluble fraction (fraction B), and water-soluble fraction (fraction C).

### 4.4. Purification of Biologically Active Constituents from Fraction A

To purify the constituents, fraction A (30.0 g) was separated by silica gel column chromatography (7.0 cm internal diameter (i.d.) × 20 cm) because this fraction exhibited the highest suppressive activity against NO production in rat hepatocytes. The compounds were eluted stepwise using *n*-hexane:EtOAc (100:0) to (0:100) to produce subfractions A1–A14. The combined weight of all subfractions was 28.7 g, and 95.5% of the starting material (30.0 g) was recovered. Subfraction A4 (5.01 g), which suppressed NO production, was purified by silica gel column chromatography using *n*-hexane:EtOAc (90:10) to (50:50) to obtain subfractions A4-1 to A4-14. Subfraction A4-6 (1.21 g) was subjected to silica gel column chromatography using *n*-hexane:EtOAc (85:15) to (65:35) and then *n*-hexane:EtOAc (85:15) to (75:25). After recrystallization in methanol, white crystals (201 mg) were obtained and designated compound **1**. Subfraction A4-5 (918 mg) was subjected to silica gel column chromatography using *n*-hexane:EtOAc (90:10) to (70:30) and then *n*-hexane:EtOAc (85:15) to (75:25). After preparative HPLC and recrystallization in methanol, white powders designated compound **2** (26.1 mg) and compound **3** (42.3 mg) were obtained. Then, the structural formula was determined from the NMR spectrum.

### 4.5. Measuring the Content of Compounds by HPLC

According to a previously published method [11], HPLC analysis was performed to measure the content. An HPLC system using an LC-20AT pump equipped with an SPD-20A UV/VIS detector (Shimadzu Corporation, Kyoto, Japan) and a Cosmosil 5C_18_ AR-II column (4.6 mm i.d. × 150 mm; Nacalai Tesque Inc.) was used. The compounds were eluted at a flow rate of 1.0 mL/min by a linear gradient (0–30 min) of a mobile phase of 0.1% formic acid in a water: methanol mixture (30:70 to 20:80) and detected at a wavelength of 322 nm. Single peaks with retention times of 7.44 min, 13.7 min, and 17.3 min corresponded to praeruptorin A, B, and E, respectively. The retention time was used to estimate the content in fraction A. Isolated praeruptorins A, B, and E were used as standards by accurate weighing and dissolution in methanol to make a stock solution of 1.00 mg/mL. Each stock solution was diluted to prepare the standard solutions at the following concentrations: 0.100, 0.200, 0.400 mg/mL (praeruptorin A); 0.0500, 0.100, 0.200 mg/mL (praeruptorin B); and 0.0500, 0.100, 0.200 mg/mL (praeruptorin E). Each standard solution (20.0 μL) was analyzed in triplicate. The calibration curve of each standard compound was calculated by plotting the peak areas (*y*) against a series of injection amounts (*x*), and the regression equation was calculated in the form *y* = A*x* + B: praeruptorin A, *y* = 17,636,470 *x* − 42,587.92 (*R*^2^ = 0.9999); praeruptorin B, *y* = 12,977,420 *x* − 105,805.6 (*R*^2^ = 0.9999); and praeruptorin E, *y* = 19,224,610 *x* − 10,635.17 (*R*^2^ = 0.9994). Fraction A was accurately weighed and dissolved in methanol to prepare a sample solution of 1.00 mg/mL, and the sample solution (20.0 μL) was analyzed in triplicate. The peak areas of compounds in the sample solution were fitted to the calibration curves, and the amounts of praeruptorins A, B, and E in 20.0 μL of the sample solution were calculated. The amounts of the compounds in 20.0 μL of the sample solution (20.0 μg of fraction A) were calculated to be 4.80 μg, 1.55 μg, and 1.81 μg. Therefore, the contents of praeruptorins A, B, and E in fraction A are 24.0%, 7.75%, and 9.07%, respectively.

### 4.6. 3D-HPLC Analysis

According to a previously published method [35], 3D-HPLC fingerprints were analyzed using an HPLC system (Shimadzu Corporation) that was equipped with a CBM-20A communication bus module, an LC-20AD binary pump, a DGU-20A3 degasser, an SIL-20AC autosampler, a CTO-10A column oven, and an SPD-M20A DAD. An Atlantis T3 column (i.d. 2.1 mm × 150 mm, 5 μm; Waters Corporation, Milford, MA, USA) was used for separation at a column temperature of 40 °C. The mobile phase was a binary eluent comprising (A) 5 mM ammonium acetate solution and (B) acetonitrile under the following gradient conditions: 0–30 min, linear gradient from 10% to 100% B; 30–40 min; and isocratic at 100% B. DAD was set up in the range of 190–800 nm, and the flow rate was 0.2 mL/min.

### 4.7. Animals and Primary Cultured Rat Hepatocytes

All animal care and experimental procedures were carried out in accordance with the laws and guidelines of the Japanese government and were approved by the Animal Care Committee of Ritsumeikan University, Biwako-Kusatsu Campus (Nos. BKC2018-029 and BKC2021-031). Specific pathogen-free male Wistar rats (5–6 weeks old; Charles River Laboratories Japan Inc., Yokohama, Japan) were housed under a 12 h light/dark cycle at 21–23 °C. The rats were fed a γ-ray-irradiated CRF-1 diet (Charles River Laboratories Japan) with water available ad libitum.

After one week of acclimatization, the livers of Wistar rats were used to prepare hepatocytes according to a previously published method [36]. Briefly, collagenase from *Clostridium histolyticum* (FUJIFILM Wako Pure Chemical Corporation) was used for the perfusion of the liver on day 0. The dispersed cells were centrifuged four times and then resuspended in Williams’ E (WE) medium supplemented with newborn calf serum (10%), dexamethasone (1 × 10^−8^ M), and human insulin (1 × 10^−8^ M). Hepatocytes were seeded at 1.2 × 10^6^ cells per 35 mm diameter dish. More than 200 dishes were usually prepared from a rat. At 2 h and 5 h after seeding, the medium was replaced by a serum-free WE medium. The purity of the hepatocytes was more than 99% by microscopic observation. The cells were incubated at 37 °C overnight (for 12 h). On day 1, the medium was replaced by a WE medium including 1 nM rat IL-1β ± fraction or compound. Each fraction or compound was dissolved in dimethyl sulfoxide, which did not affect NO production when its concentration was less than 1% (*v*/*v*) in the medium [37]. The hepatocytes were further incubated at 37 °C for 4 h before total RNA extraction or for 8 h before the NO levels were measured and the cell lysates were prepared for Western blotting.

### 4.8. Rat IL-1β

The cDNA for the mature peptide (amino acid residues 117-268) of rat IL-1β (DDBJ/EMBL/GenBank accession No. NM_031512) was amplified by PCR using the primers 5’-CCAGCATATGGTTCCCATTAGACAGCTGCACTG-3’ and 5’-CCGAATTCAGGAAGACACGGGTTCCATGG-3’. The amplified DNA fragment was digested with Nde I and EcoRI and cloned into the pCold III vector (Takara Bio Inc., Kusatsu, Shiga, Japan). The resultant construct and pLysSRARE2 plasmid (Novagen Inc., Madison, WI, USA) were introduced into *Escherichia coli* strain BL21 (Takara Bio Inc.). The transformed cells were grown and cold-shocked. The cells were harvested, lysed, and subjected to ammonium sulfate precipitation and dialysis, followed by ultrafiltration to purify the mature peptide, the activity of which was verified by comparison with that of recombinant rat IL-1β (PeproTec, Rocky Hill, NJ, USA).

### 4.9. RT–qPCR

After 4 h incubation of hepatocytes with 1 nM rat IL-1β ± a compound on day 1, total RNA was prepared from hepatocytes using Sepasol I Super G solution (Nacalai Tesque, Inc.) [4,8]. The cDNA was reverse-transcribed from total RNA and amplified by PCR with the primers described in Table 3. By real-time PCR using SYBR Green I and the Thermal Cycler Dice Real Time System (Takara Bio Inc.) or the Rotor-Gene Q 2plex HRM System (Qiagen, Hilden, Germany), the mRNA levels were quantitatively measured in triplicate. We confirmed a single peak of melting temperature on the dissociation curve of the amplified product from each mRNA. Then, the threshold cycle (Ct) value was used for the subsequent calculation. According to the ΔΔCt method, the relative mRNA levels were calculated from these obtained Ct values. The Ct values were normalized to *EF-1α* mRNA [4,8], which is encoded by a housekeeping gene (an internal control). The normalized mRNA levels in the total RNA from IL-1β-treated hepatocytes were set at 100%.

### 4.10. NO Assay and LDH Activity

After 8 h incubation with IL-1β on day 1, the hepatocytes were incubated with 1 nM IL-1β ± a fraction or compound for 8 h. Nitrite, which is a stable metabolite of NO, in the medium was measured in triplicate by the Griess method [3,38]. The NO concentration in the medium alone was set as 0%, and the concentration in medium including IL-1β was set as 100%. The IC_50_ values of nitrite were calculated for three different concentrations of a fraction or a compound unless it showed cytotoxicity [3]. Loxoprofen sodium (Kolon Life Science, Inchon, Republic of Korea) was used as a control. Cytotoxicity to hepatocytes was assessed by estimating LDH activity in the medium using an LDH Cytotoxicity Detection Kit (Takara Bio Inc., Otsu, Japan). LDH activity of a whole cell extract was assumed to be 100%. The medium was mixed with Griess reagent [3,38] and incubated at 20–23 °C for 5 min, and absorbance at 540 nm was measured to determine the decrease in nitrite induced by the compound.

### 4.11. Western Blot Analysis

Western blotting was performed according to a previously described method [5]. Briefly, hepatocytes were incubated with 1 nM IL-1β ± fraction or compound for 8 h. Cell lysates were prepared in the presence of a protease inhibitor cocktail (Nacalai Tesque, Inc.). The resultant lysates were electrophoresed on a 10% sodium dodecyl sulfate-polyacrylamide gel and transferred onto a Sequi-Blot membrane (Bio-Rad, Hercules, CA, USA). After blocking with 5% Difco skim milk (BD Biosciences, San Jose, CA, USA), target proteins were immunostained using primary antibodies against iNOS (BD Biosciences) and β-tubulin (Cell Signaling Technology Inc., Danvers, MA, USA), followed by horseradish peroxidase-conjugated anti-immunoglobulin Fc antibody. The protein was visualized with Enhanced Chemiluminescence Blotting Detection Reagents (GE Healthcare Biosciences Corp., Piscataway, NJ, USA) and detected using an Amersham Imager 600 (GE Healthcare)

### 4.12. Statistical Analysis

The data are representative of at least three independent experiments that provided similar findings. The values are shown as the mean ± SD. The differences were analyzed using Student’s *t* test followed by the Bonferroni correction. The significance was set at *p* < 0.05 and *p* < 0.01.

## 5. Conclusions

Principal constituents were isolated from a *P. praeruptorum* root extract, and NO production in IL-1β-treated rat hepatocytes was monitored to guide the isolation procedure. As a result, praeruptorins A, B, and E were identified, and praeruptorin B showed the highest potency to inhibit NO production by inhibiting the expression of the *iNOS* gene, as well as proinflammatory genes. These three pyranocoumarins may primarily contribute to the anti-inflammatory effects of *P. praeruptorum* roots.

## Figures and Tables

**Table 1:** Fractionation of a *P. praeruptorum* root extract and its effects on NO production in hepatocytes.

Extract/Crude Fraction	Yield [%] ^1^	IC_50_ [μg/mL] ^2^
Methanol extract	100.0	154.3 ± 26.8
Fraction A (EtOAc-soluble)	29.3	24.1 ± 3.0
Fraction B (*n*-butanol-soluble)	8.76	74.4 ± 27.3
Fraction C (water-soluble)	62.0	NA

^1^ The percentage of the weight was calculated by dividing each fraction by the sum of three fractions. ^2^ The value (mean ± SD) was calculated by at least three independent assays showing similar findings. NA: not applied due to low activity.

**Table 2:** Effects of constituents in fraction A of *P. praeruptorum* root extract on NO production in IL-1β-treated hepatocytes.

Constituent (Compound)	Content [%] ^1^	IC_50_ [μM] ^2^
Fraction A	100.0	––
Praeruptorin A (**1**)	24.0	208 ± 77.3
Praeruptorin B (**2**)	7.75	43.5 ± 7.54
Praeruptorin E (**3**)	9.07	57.4 ± 7.00

^1^ The content of each constituent was measured using HPLC and is shown as the percentage of dry weight of fraction A. ^2^ The value (mean ± SD) was calculated by at least three independent NO assays.

**Table 3:** Pairs of primers used for PCR: forward (upper) and reverse (lower) primers.

Name ^1^	Gene ^2^	Primers (5’-to-3’ Direction)
Albumin	*Alb*	TGACAAGTCCATTCACACTCTCT
		AACTTCATGCAAATAGTGTCCCAG
CCL20	*Ccl20*	CAGCCAGTCAGAAGCAGCAAGC
		CTTGGTTCTTAGGCTGAGGAGGT
EF-1α	*Eef1a1*	TCTGGTTGGAATGGTGACAACATGC
		CCAGGAAGAGCTTCACTCAAAGCTT
GR	*Nr3c1*	CAAAGGCGATACCAGGCTTCAGAA
		GAGACATTCTCTGCTCATTAATAATC
IL1R1	*Il1r1*	GTTAGTATACCCAGTTCCTGACT
		GTCTTTCCATCTGAAGCTTTTGG
iNOS	*Nos2*	CCAACCTGCAGGTCTTCGATG
		GTCGATGCACAACTGGGTGAAC
Insr	*Insr*	CTTCCGACATGTGGTCCTTTGG
		TCAGGTCAGTGAGTCTCTCTGGA
TNF-α	*Tnf*	TCCCAACAAGGAGGAGAAGTTCC
		GGCAGCCTTGTCCCTTGAAGAGA

^1^ Abbreviated names of proteins. ^2^ Gene symbols are indicated. *EF-1α*, *iNOS*, and *GR* mRNAs are used instead of gene symbols.

## Data Availability

The data presented in this study are available on request from the corresponding author.

## Sample Availability

Samples of the compounds are available from the corresponding author unless their amounts are very few.
